# Toward Safe and Confident Silver Drivers: Interview Study Investigating Older Adults’ Driving Practices

**DOI:** 10.2196/57402

**Published:** 2024-08-12

**Authors:** Sunyoung Kim, Phaneendra Sivangula

**Affiliations:** 1 Department of Library and Information Science Rutgers University New Brunswick, NJ United States

**Keywords:** older adults, driving, transportation, healthy aging, aging in place, quality of life

## Abstract

**Background:**

As the aging population in the United States continues to increase rapidly, preserving the mobility and independence of older adults becomes increasingly critical for enabling aging in place successfully. While personal vehicular transport remains a popular choice among this demographic due to its provision of independence and control over their lives, age-related changes may heighten the risk of common driving errors and diminish driving abilities.

**Objective:**

This study aims to investigate the driving practices of older adults and their efforts to maintain safe and confident driving habits. Specifically, we sought to identify the factors that positively and negatively influence older adults’ driving performance and confidence, as well as the existing efforts put into sustaining their driving abilities.

**Methods:**

We recruited 20 adults aged ≥65 years who remained active drivers during the recruitment from the greater New York area. Then, we conducted semistructured interviews with them to examine their perceptions, needs, and challenges regarding safe and confident driving.

**Results:**

Our findings uncovered a notable disparity between older adults’ self-perceived driving skills and the challenges they face, particularly caused by age-related limitations and health conditions such as vision and memory declines and medication routines. Drawing on these findings, we proposed strategies to bridge this gap and empower older adults to drive safely and confidently, including fostering a realistic understanding of their capabilities, encouraging open dialogue regarding their driving, encouraging regular assessments, and increasing awareness of available resources.

**Conclusions:**

This study uncovered a noticeable disparity between the perceived driving competence of older adults and the actual challenges they confront while driving. This divergence underscores a significant need for better support beyond the existing aid available to preserve older adults’ driving skills. We hope that our recommendations will offer valuable insights for practitioners and scholars committed to enhancing the overall well-being and quality of life for older adults as they age in their homes.

## Introduction

### Background

Due to the culture, lifestyles, and vast geography of the United States, automobiles play a crucial role in enhancing the quality of our daily lives by serving as the key to mobility, freedom, and independence. Driving holds particular significance for older adults in today’s aging society, as it enables them to maintain their socialization, independence, and mobility as they age [1-3]. The absence of driving can lead to isolation and depression among older adults [4], with consequent declines in both their physical and mental health [5-7]. Therefore, it becomes important to ensure that these drivers maintain and prolong their driving skills for independence, healthy aging, and overall quality of life.

According to the American Automobile Association, drivers aged between 60 and 69 years are considered the safest drivers in the United States [8]. Even drivers aged ≥80 years are involved in fewer car accidents per mile driven than younger drivers [9]. Furthermore, drivers in their 70s have lower rates of police-reported crash involvements per capita than middle-aged drivers [9]. Consequently, older adults can significantly contribute to our driving culture as some of the safest drivers. However, age-related changes can adversely affect the driving abilities of older individuals [10,11]. For example, as individuals age, they may experience shifts in reaction time, visual acuity, and cognitive processing, which can impair their capacity to drive safely [12,13]. Therefore, it is crucial for older adults to recognize that aging may increase the risk of safe driving and that they must be vigilant about signs indicating such changes and make efforts to maintain their driving capabilities.

Existing efforts to help maintain older adults’ driving capabilities encompass programs focused on driver training, self-assessment tools, and regular health checkups [12,14,15]. However, little is known about the efforts that older drivers exert to cope with their aging-related changes and maintain their driving capabilities, let alone how they use any of these existing programs.

This study seeks to comprehend the current practices associated with daily driving experiences among older adults, providing insights into actionable and empirical implications to help older adults sustain their driving capabilities. Through semistructured interviews with 20 participants aged ≥65 years who were actively driving, we explored their perceptions of their driving abilities, identified the challenges they face while driving, examined strategies for managing these challenges, and assessed the efforts they put into maintaining their driving capabilities, if any.

Our findings revealed a significant disparity between the confident mindset that the participants held regarding their driving abilities and the challenges they encountered while driving. Although most participants expressed no immediate concern or effort about maintaining their driving proficiency and displayed overall confidence in their current capabilities, they acknowledged various challenges that negatively impacted their driving performance and confidence only when prompted about specific driving-related challenges. This highlights a significant disparity between the perceived need for support and the actual assistance required to uphold driving performance among older adults.

In what follows, we describe a review of existing related works, our study procedure, and key findings. We conclude this paper by discussing the implications for supporting the maintenance of older drivers’ driving capabilities, along with addressing some limitations of this study.

This study aims to investigate the driving practices of older adults and their efforts to maintain safe and confident driving habits and to explore strategies that help older adults continue driving safely and confidently.

### Literature Review

#### Driving and Quality of Life in Older Adults

The World Health Organization recognizes that maintaining mobility is fundamental to active aging, enabling older adults to remain engaged in various activities [16]. Loss of mobility, often stemming from the inability to operate a vehicle safely, can lead to activity restriction, physical deconditioning, and reduced social participation, significantly affecting an individual’s health and overall quality of life [17]. In particular, impaired mobility has been identified as an early predictor of physical disability [18] and is associated with negative outcomes such as falls, loss of independence, institutionalization [19], and even mortality among older adults [20].

Undoubtedly, 1 key aspect of mobility for older adults is maintaining their driving privileges [21]. It is evident that the stage at which individuals change their driving behavior depends on various internal and external factors, such as personal circumstances and declining abilities [22]. Some individuals may abruptly cease driving due to external pressures or sudden events, while others who gradually restrict their driving likely do so in response to internal factors related to their abilities [21,23]. Those who prepare for driving cessation often have a combination of internal and external factors guiding their decision-making [24]. Research also highlights the profound impact of driving cessation on older adults’ well-being. Several studies linked driving cessation to increased depressive symptoms [25,26] and identified it as a predictor of mortality risk [27].

Especially for many older adults in rural areas, driving remains critical for maintaining independence [8]. It aids daily living activities, supports familial responsibilities, and promotes community integration [23]. Even when facing physical and cognitive declines that may limit their ability to drive safely, some older adults continue to drive, as it contributes significantly to their self-confidence, well-being, and social connections [23]. As such, the relationship between driving and the quality of life in older adults is a critical aspect of aging, as it directly impacts their ability to lead dynamic and independent lives.

#### Physical, Cognitive, and Social Factors in Older Adults’ Driving Capabilities

The overall physical health has been acknowledged as a crucial factor impacting older adults’ ability to drive and respond effectively to unforeseen situations on the road [28]. Numerous studies have documented the correlation between health issues and the driving capabilities of older adults [2,23,29,30]. Generally, the findings indicate that health problems tend to increase with age, placing individuals with various health issues at a heightened risk for diminished driving performance. Specifically, vision impairments and physical limitations are found to be strongly associated with driving performance, albeit to varying degrees [31].

Cognitive abilities, including attention, memory, and executive function, are also critical for quick decision-making and multitasking, both essential for safe driving [28]. Several studies have underscored the crucial role of cognitive and neuropsychological factors in evaluating older adults’ driving capabilities. Factors such as processing speed, divided attention, visuospatial abilities, executive functions, psychomotor speed, and overall cognitive functioning have emerged as pivotal predictors of safe driving among older adults [31-33].

Social factors such as retirement and personal loss have also been found as triggers for driving anxiety among older drivers, thereby influencing their driving behavior [12,34]. These social circumstances can have immediate and long-term impacts on driving capabilities, highlighting the importance of preventive measures and interventions to address them [25].

Although age-related declines occur, older adults may not be aware of these changes or how they affect driving. Some older drivers fail to recognize their declines or overestimate their driving safety [24,25]. In this context, self-regulation and self-awareness play pivotal roles in how older adults adapt to these challenges, ultimately shaping their decisions and behaviors related to driving. Therefore, it is crucial to assist older adults with lower levels of health risk in increasing awareness of their health status concerning driving capabilities and taking preventive actions to maintain their ability to drive safely.

#### Existing Efforts to Help Maintain Older Adults’ Driving Skills

A systematic review of driver-training interventions for individuals aged ≥55 years, without any health-related impairments that prevent licensure, showed that interventions tailored to individual participants’ health conditions can positively impact driving ability and on-road performance [35]. Compared to the extensive studies on understanding factors that affect older adults’ driving performance, relatively few studies have explored the impact of tailored interventions on sustaining the driving safety of older adults. One exception is a randomized controlled trial that found tailored driving lessons reduced critical driving errors among older adults, acknowledging the need for longer-term follow-up and more extensive trials [28]. In addition, cognitive training, focusing on speed of processing and reasoning skills, demonstrated promising results, with a lower at-fault motor vehicle collision rate observed in older drivers who received training than controls [36]. These findings highlight the potential of personalized training interventions to enhance driving safety, sustain independence, and improve the quality of life for older adults, given the significance of driving mobility and the associated costs of declining driving capabilities.

In addition, driving simulator training has emerged as a useful tool to enhance driving skills in older adults for its effectiveness of attention training [24]. The implications of these interventions are found to be substantial in improving older drivers’ safety on the road [20]. Given the variability in older adults’ driving experiences, skills, and self-awareness, researchers argue that the strategies to help maintain older adults’ driving skills must be customized to address individual strengths and areas for improvement [37].

By delving deeper into the daily driving practices and concerns among older drivers, this research seeks to understand the complex interplay between aging, the challenges associated with driving, and the strategies to maintain mobility and safety. Through this investigation, the study aims to offer insights that can inform future interventions and policies to support older adults’ driving needs.

## Methods

### Ethical Considerations

The study was approved by the Rutgers Institutional Review Board (Pro2023001102), and informed consent was obtained from all participants before participating in the study. At the end of the interview, each participant was duly compensated with a US $30 Amazon gift card in recognition of their time and willingness to participate in the study.

### Participant Recruitment

The recruitment of participants followed a systematic process aimed at ensuring a representative and ethically sound sample. To identify potential participants, the first author visited local libraries, senior centers, and low-income living facilities situated in the vicinity of the greater New York area. During our engagement with these facilities, the study’s objectives and purpose were comprehensively elucidated to the respective managers and supervisors. Upon their explicit approval and support, recruitment flyers were prominently displayed in the facility lobbies and on designated advertising boards.

The eligibility criteria for participant recruitment encompassed 3 primary requirements. First, participants had to be aged >65 years. Second, they needed to possess a valid US driver’s license, demonstrating prior driving experience. Third, they needed to be engaged in driving as part of their daily routine. This methodical recruitment approach ensured that our sample consisted of individuals who met the age criterion, had a documented driving history, and were healthy enough to sustain driving.

We recruited 20 participants, all of whom boasted substantial driving experience and maintained their active status as drivers (mean driving experience 51.1, SD 8.54 years). A gender distribution of approximately 70% (14/20) of female participants and 30% (6/20) of male participants was observed within the sample. The average age of our participants was 71.7 (SD 5.81) years. A breakdown of participant demographics is provided in Table 1 for further insight.

**Table 1:** Participant demographics and study duration (some participants included their learner’s permit periods in their years of driving experience; N=20).

ID	Age (years)	Gender	Driving experience (years)	Approximate driving frequency	Medical condition
P1	77	Woman	58	2 to 3 times a week	None
P2	65	Woman	50	Every day	None
P3	82	Woman	61	4 to 5 times a week	Cataract surgery
P4	77	Man	59	2 to 3 times a week	None
P5	73	Woman	50	Every day	None
P6	66	Woman	48	Every day	Survivor of breast cancer
P7	76	Woman	59	Every day	None
P8	73	Man	57	5 times a week	None
P9	83	Woman	59	2 to 3 times a week	None
P10	69	Man	52	4 to 5 times a week	Cardiac rehabilitation
P11	68	Man	32	Every day	None
P12	70	Man	52	Every day	None
P13	69	Woman	49	Every day	None
P14	73	Woman	55	Every day	None
P15	65	Woman	30	4 to 5 times a week	None
P16	67	Man	40	2 to 3 times a week	None
P17	74	Man	50	Every day	None
P18	84	Woman	60	5 to 6 times a week	None
P19	74	Woman	37	Twice a week	None
P20	68	Woman	40	Every day	None

### Data Collection

The research methodology used in this study was carefully designed to investigate the perspectives that older adults had with their daily driving experiences, as well as the challenges faced by them in maintaining their driving capabilities.

A comprehensive interview protocol was crafted to facilitate in-depth discussions with the study participants. The protocol comprised a series of open-ended interview questions, ensuring a flexible and exploratory approach to data collection. The questions were purposefully designed to elicit a broad range of responses, enabling a comprehensive understanding of the participants’ experiences and perspectives.

The data collection process involved conducting in-person, semistructured interviews with each participant. These interviews were scheduled at a location of the participants’ preference to ensure their comfort and convenience. This approach facilitated candid and open conversations, allowing participants to express their thoughts and experiences freely. In addition to the interview responses, essential demographic information was gathered from each participant. This information encompassed details such as age, gender, prior health issues, and their level of familiarity and experience with technology. These demographic factors were considered in the data analysis to provide a comprehensive context for the study.

To maintain the integrity of the research, great care was taken to avoid any form of bias in the interview process. The questions posed during the interviews were constructed in response to the participants’ narratives and experiences, ensuring that the research did not unduly influence or guide their responses. This approach aimed to collect data that were a true reflection of the participants’ perspectives and challenges.

The duration of each interview session ranged from 30 minutes to 1 hour, depending on the depth and complexity of the participants’ experiences and responses. This flexible approach allowed for comprehensive data collection without imposing undue time constraints on the participants.

To ensure accuracy and a thorough analysis, all interviews were audio recorded. These recordings were later transcribed to create a written record of the interviews, which served as the basis for data analysis and the development of research findings.

### Data Analysis

We used thematic analysis to identify and understand patterns across our interview data [38,39]. We began by open coding the data, which involved identifying and labeling key concepts. We then used axial coding to group related concepts into broader categories, which we called phenomena. Phenomena are recurring events, actions, and interactions representing people’s responses to problems and situations. Finally, we used selective coding to assemble the phenomena into a cohesive framework. We iterated all the steps until we achieved data saturation, signifying our thorough exploration of themes and patterns within the interview data. The goal of this step was to understand the relationships between the different phenomena and develop a comprehensive understanding of the interview data.

## Results

### Overview

The prevailing sentiment among our participants regarding their driving abilities was confidence in their abilities as good drivers. Consequently, a majority did not express any need or support for sustaining their driving proficiency, leading to consent among participants that such support was unnecessary. However, when probed about challenges related to their driving, several health-related issues emerged as factors adversely affecting their driving performance as well as their driving confidence. This dissonance between a confident mindset about driving and the escalating challenges encountered while driving among older drivers contributed to a significant gap between the perceived need for support and the actual assistance required to maintain their driving performance. Next, we first described how our participants perceived their current driving performance and the factors that positively contributed to their confidence in driving. Then, we reported a range of challenges that negatively affected driving performance. Finally, we concluded this section by describing the efforts aimed at maintaining driving capabilities. See Textbox 1 for a summary of our findings.

**A summary of themes and factors.**

**Factors positively contributing to older adults’ driving confidence**

- Active and independent driving
- Long-term, adaptive driving experience
- Physical fitness

**Challenges older adults face in driving**

- Vision challenges
- Memory decline
- Medication routines

**Efforts to sustain driving**

- Physical exercise
- Technology support: navigation systems
- Keeping focus undistracted while driving

### Factors Positively Contributing to Driving Confidence

#### Active and Independent Driving

All participants reported that they were competent in their current driving skills. Several participants even expressed their pride in engaging in driving actively and regularly:

Driving is not a botheration for me at all. I would say 95% of the time, people want me to drive it because they feel I drive well and defensively.

Participant 17

I enjoy driving. It gives independence rights. I can go anywhere.

Participant 11

Participants mentioned several factors positively contributing to their belief that they were skilled and capable drivers.

As shown in Table 1, our participants exhibited a consistent trend of active driving, engaging in daily or near-daily driving activities. Daily routines involve driving for various purposes, including grocery shopping, medical appointments, and visits to family and friends. While local travel, covering short to moderate distances, was standard, participants also undertook occasional long-distance drives for special events or trips. Then, they expressed that these regular journeys contributed to their confidence in their driving skills. They attributed their confidence to the notion that by continuously driving, they were continually honing their abilities. The more they drove, the better they perceived themselves to be. For many, having a car and using it as their primary mode of transportation were testaments to their ongoing commitment to maintaining their driving skills:

I think driving skills improve on how often you drive. If you don’t drive, then you never get improved. If you keep driving, then you will improve, and keep improving.

Participant 18

I have a car, I drive everywhere. And as far as possible, if I have to go anywhere within a certain radius, I prefer to drive rather than take public transportation.

Participant 17

Participants proudly emphasized their decades of solo travel on the road. This wealth of experience has undoubtedly contributed to a heightened sense of self-assuredness in their driving capabilities. These experiences, often marked by daily commutes, long-distance journeys, and even the foundational act of obtaining a driver’s license, imbued them with a deep-seated belief in their driving proficiency:

I live alone. I gotta drive by myself. Driving myself is more accessible than using public transportation.

Participant 20

I drove from here to Dallas, and then back and forth a couple of times. Earlier, I used to drive for eight hours every day. So, I can drive with no problem.

Participant 20

When I was working, I used to get up early in the morning have a quick shower, and then drive down to work for the most part in my working career, I drove in excess of 50 miles every day.

Participant 17

Participants expressed that their ability to maintain their daily routines, even amid health challenges, reinforced their confidence in their driving skills. Whether running errands or embarking on longer trips, the participants frequently reiterated that they were accustomed to traveling alone. As such, this sense of independence in driving solo, sometimes despite challenges, strengthened their confidence in driving as skilled and independent drivers:

I’m good now. Yeah. I mean, I went through eight weeks of radiation. I drove myself every day. And that was not a problem.

Participant 14

My cataract surgery didn’t go well. The doctors didn’t correct my eyesight properly in my right eye. I use monocular vision. When the car gets too close, I will switch to my left eye by closing my right, allowing me to continue driving despite the challenge.

Participant 3

#### Long-Term, Adaptive Driving Experience

Our participants have driven for 51 (SD 8.54) years on average. This long-term period of driving contributes to fostering confidence to navigate the road effectively. Furthermore, these experiences have instilled a sense of resilience and adaptability, making them feel that driving in comparatively more spacious and less-congested locations is a breeze. This adaptability also positively contributed to their confidence in driving, as their driving skills are up to par, irrespective of the location:

I have traveled alone for the last 40 years.

Participant 1

I’m 77, and I got my driver’s license when I was 18. So, I have been driving for 59 years.

Participant 18

I came to the United States for my graduate study. That’s 1986. And I bought a used car. And I started driving regularly.

Participant 20

In particular, extensive driving experiences in different geographical characteristics positively influenced the development of adaptive driving experiences among our participants. Participants shared that they had experienced challenging driving environments throughout their lifelong driving experiences, such as driving along the bustling streets or the narrow and crowded roads of the city countless times. Participants expressed how their initial driving experiences in their countries of origin were marked by traffic congestion, narrow roads, and limited parking spaces. Their proficiency in handling diverse driving environments, from lifelong driving experiences in congested cities to more open rural areas, had bolstered their self-assuredness in driving:

Once you overcome that by driving in New York, you feel it’s just a cakewalk everywhere driving.

Participant 6

I have no problem driving anywhere, no matter what kind of place it is, whether it’s crowded or not because I have driven in India.

Participant 16

Driving is okay because you know, in Taiwan, the traffic is so bad. And the roads are so narrow. And the parking space is narrow. Up here all the roads are much bigger, and the parking space is bigger too. So, for me, it’s easy.

Participant 20

#### Physical Fitness

The prevailing sentiment among our participants about their health conditions was positive. Participants said that their physical health remained in relatively good shape and did not mention any significant health issues. While some participants mentioned minor challenges in their physical condition, such as feeling weaker or less energetic, all of them said that these challenges did not hinder their driving capabilities much. Only a couple of participants shared specific health concerns, such as vision problems due to unsuccessful cataract surgery, or concerns relating to survivors of cancer or those undergoing cardiac rehabilitation. Unsurprisingly, therefore, we found that participants’ good health conditions and physical fitness positively affected their perception of being adept drivers. As such, the absence of health issues and mobility challenges contributed to building a sense of self-confidence about driving skills:

At the moment, I feel comfortable driving and I don’t have any health conditions. That should not be a problem for me driving.

Participant 8

I can see that my heart lot better after I retired because doing activities and stuff like that. Before I used to work longer hours and I was always tired. But now I have a lot of energy and I can do myself a lot of different things.

Participant 15

In addition, a positive perception of overall health conditions led them to believe that their driving abilities could endure for a considerably prolonged period. They associated good health and overall energy levels with their potential to drive effectively, even after retirement:

I think I still can drive for the next 20 years because I always believe we have good genes because my dad’s life is 100.

Participant 20

Serious conditions like diabetes or any other conditions may disturb driving, but regular vision or something muscular isn’t stopping driving.

Participant 3

### Challenges Faced in Driving

#### Overview

While our participants generally held positive perspectives regarding their driving capabilities, a contrasting reality emerged when we delved into the challenges that they encountered while driving. Participants reported a range of challenges stemming mostly from individuals’ health conditions and physical declines due to aging, which echoes much prior work [2,19,20,31,32,40,41].

#### Vision Challenges

Among the challenges frequently highlighted by the participants, aging-related vision issues emerged as a prevalent concern. These included conditions such as nearsightedness, presbyopia, sensitivity to light and glare, and cataracts. The aging process often introduces visual impairments that might significantly impact safe driving [19,41]. Participants acknowledged the obstacles posed by these aging-related vision issues, even when using corrective eyewear. In particular, night driving emerged as a significant challenge for our participants due to reduced visibility caused by inadequate lighting and distracting glares. This discomfort significantly contributed to having a fear of driving at night among our participants. The disruptive effect of the lights from incoming cars was a recurring theme, with participants describing it as “very difficult” and sometimes “blinding.” Consequently, participants stated that they voluntarily restricted their driving activities to daytime hours and, in emergencies, reluctantly ventured out at night:

I thought I just needed a new eyeglass prescription (for driving? Or in general?), but apparently, I was starting to develop a cataract, so I’m not comfortable driving at night.

Participant 10

When I drive during the night, it looks like the light from across the car is coming too quickly. It’s as if you cannot see, and you are suddenly blinded.

Participant 15

I can see clearly in the morning. Probably my, my eyes are aging for the night. I don’t drive at night.

Participant 13

While various vision changes can influence driving abilities across different age groups, cataracts were mentioned as a significant concern that is directly connected to aging. Participants mentioned issues relating to the development of cataracts as they aged, often noting that they might not immediately recognize their onset:

The only issue I have with driving now is night driving, and it’s a curious issue. I had a cataract, and I went for surgery, but it didn’t go well.

Participant 3

#### Memory Decline

We confirmed that memory decline in older adults is another critical factor significantly influencing their ability to drive safely and confidently. Participants mentioned how memory decline has negatively impacted their driving experiences and heightened concerns about their safety on the road. They expressed a general feeling of reduced alertness in older age compared to their younger years. Although they lacked quantitative data to confirm a decline in their reaction time, they acknowledged a perceptible change in their level of alertness while driving. This sense of reduced alertness can significantly affect their ability to respond promptly to unexpected events on the road, potentially compromising safety, which echoes prior work [35]:

I feel that reaction time is slower. But I haven’t tested my reaction time.

Participant 3

One recurrent concern regarding memory decline among participants was a diminished awareness of their surroundings, particularly when changing lanes or responding to vehicles approaching from the side. Memory lapses were particularly concerning at intersections and stop signs. Participants admitted to being more forgetful when it came to looking both ways and ensuring that there were no oncoming cars. Such lapses in memory can lead to critical oversights and increased risk at intersections, which are the common sites for accidents:

I’ve become a little less likely to notice a car that’s coming up on one side while I’m about to change lanes. Also, when I come to a stop sign, I’m a little bit more forgetful to look both ways and make sure there are no cars coming. I don’t have any quantitative evidence that there’s a decline in my reaction time, but I just don’t feel quite as alert as I did 10 or 20 years ago.

Participant 8

#### Medication Routines

Participants mentioned the increasing need for taking medication in their daily routine as a factor negatively influencing their driving abilities. As they confronted more health issues as they aged, participants relied on a more complex regimen of medications to manage various ailments. In particular, we identified the necessity for our participants to take multiple medications daily, often comprising pills for conditions such as high blood pressure, high cholesterol levels, and heart problems. These medications, while vital for health management, can lead to immediate physical discomfort and limitations. For instance, certain blood pressure medications can cause a significant drop in blood pressure upon ingestion, rendering individuals unfit to drive for a specific period, typically an hour. This medication routine apparently translated into challenges and constraints regarding safe driving and might necessitate meticulous planning around medication schedules, often hindering spontaneous travel. Participants emphasized the importance of understanding these risks and the necessity for stringent precautions to prevent any accidental mishaps on the road:

I do have heart problems. I take about six pills in the morning. Because some of them are for blood pressure. And then I take three pills at night. So, when you first take the blood pressure pills, they lower your blood pressure very strongly. So, you’re not supposed to drive for like an hour.

Participant 10

I have a friend who takes insulin every day; when insulin is shot, you go into a coma, and you cannot control that car, then I think you really should be clear about driving.

Participant 18

### Efforts to Sustain Driving

#### Overview

The participants highlighted the challenges that adversely affected their driving performance; however, most of them did not exert much effort to overcome or address these challenges. Some participants mentioned that they were not aware of any available resources or support to remedy the challenges, while others expressed the belief that they did not need any assistance:

I didn’t even know something like the resources existed. So, I never thought of that. Is there anything like that?

Participant 16

Only a few participants mentioned that they had adopted efforts to maintain their independence, mobility, and safety on the road. They exhibited resilience and resourcefulness in addressing the obstacles posed by health conditions, medication, and aging-related discomfort. The efforts included doing physical exercise, using technology support for navigation, and keeping undistracted focus while driving.

#### Physical Exercise

Physical exercise emerged as a crucial strategy among our participants to counteract the physical limitations associated with aging and health issues that negatively influence their driving abilities. Participants emphasized the positive influence of regular physical activity on their overall health and specifically on their driving ability. For them, the incentive to exercise stemmed from health-related wake-up calls, such as heart attacks, muscular pains, and so on. These experiences prompted them to adopt a proactive approach to their well-being. Participants acknowledged that engaging in physical activities, such as doing physical exercises and playing sports, vastly helped maintain their driving capabilities, as it improved reflexes and hand-eye coordination. As such, the connection between physical exercises and driving was emphasized as an avenue for honing the reflexes and cognitive skills required for safe and efficient driving:

Many times, the number of games you play helps you to be reflexive, and in my case, I play sports that reflect it. It’s hand-eye coordination and also keeps you fit. This helps me to drive better.

Participant 18

That’s why I’m trying to do a lot of exercise. That’s where my muscles don’t get lost and get stronger and stronger. We have a good Senior Center exercise program over there. That helped me a lot.

Participant 15

Importantly, some participants found community resources invaluable in their quest for physical fitness. Senior center exercise programs were particularly commended for providing structured and accessible opportunities for exercise. These programs offered guidance, camaraderie, and a supportive environment that motivated older adults to stay active:

I’m trying to do a lot of exercise. We have a good exercise program at the senior center. That helped me a lot.

Participant 15

#### Technology Support: Navigation Systems

One of the most remarkable and transformative aids in the driving endeavor has been the adoption of GPS and navigation systems. This technological innovation has become an indispensable tool, significantly enhancing the safety and confidence of not only our participants, older drivers, but all individuals behind the wheel. Participants expressed a heavy reliance on GPS devices and navigation systems to bolster their driving capabilities. Some participants were using traditional stand-alone GPS devices, considering them to be highly reliable, while others were using a mobile navigation app, such as Google Maps:

I have a very old navigator. I still use that to navigate. I plug in that older one. It’s kind of more reliable.

Participant 13

I can use Google Maps and Waze maps, just to feel confident, and I have GPS on my car also. I always put GPS on my car if I had to go someplace, even my sister’s house.

Participant 15

Before starting the journeys, participants described their predrive routine, which often includes a preliminary check of routes and traffic conditions using Google Maps or other mobile mapping services. This proactive approach gives them a sense of preparedness and certainty about their route, reducing the stress of driving in unfamiliar locations:

Before I drive to a place, I prepare in advance; I use Google Maps at home and on the computer.

Participant 10

Participants emphasized that the integration of GPS functionalities into modern vehicles has been a game changer. These systems not only presented navigation instructions and route information but also provided a wealth of supplementary data about the car’s performance and its surroundings. This additional information enriched the overall driving experience, addressing sensory limitations by offering extra visual cues and audible directions. For example, signage recognition on the dashboard, even when exterior signs might be challenging to discern, provided an additional layer of safety information and reassurance to our participants:

Even though I cannot see the sign outside, I’m able to see it on the map in my dashboard, and then it tells me, like the stop signs, exits.

Participant 16

Now you can get a whole display. And you get so much more information about your car than you ever could before.

Participant 3

While these technical innovations are not exclusively advantageous for older drivers, our participants undeniably derived significant benefits from using these technologies, which enhanced their driving performance.

#### Keeping Focus Undistracted While Driving

For many of our participants, driving has become a singular task that demands their full attention. Since the act of driving itself has its inherent cognitive demands, our participants considered remaining mentally agile crucial for maintaining driving capabilities. Therefore, to focus solely on the road while driving, some participants deliberately minimized distractions by actions such as turning off the radio or refraining from engaging in lengthy conversations with passengers. They consider this a necessity, recognizing that their vigilance on the road is a key factor in their continued safe mobility. This deliberate choice reflects their understanding that maintaining their attention span while driving is crucial for their driving performance and safety:

I don’t turn the radio on. Attention spans should go to driving. And I have other things like even passengers talking to me. I tell them, you have your conversations. I have mine because I must pay attention to the road.

Participant 13

The more you talk, the more active you are, yeah, otherwise I can fall asleep.

Participant 16

## Discussion

### Principal Findings

Our findings demonstrate a significant disparity between self-perceived driving competence among our participants and the challenges they currently face, which are adversely affecting their driving abilities. Our participants consistently expressed confidence in their driving abilities, either contrasting with or regardless of the actual aging-related difficulties. While it is encouraging that older drivers maintain this confidence, the negative consequences of these perceptual gaps are far-reaching and can directly affect driving safety.

The lack of awareness or denial of their limitations can increase the risk of accidents and compromise overall driving safety [34]. For example, when older adults underestimate the significance of their vision challenges or believe that their adaptability can compensate for memory lapses, they may forego necessary medical evaluations and interventions crucial for their safety on the road [1]. Furthermore, the overreliance on a perception of good health and physical fitness can lead older drivers to disregard the potential effects of aging-related physical changes on their driving skills. This can result in underestimating the risks associated with physical discomfort and the need for medication. Therefore, this gap needs to be addressed adequately to help older adults maintain their ability to drive safely and confidently for longer periods.

The self-assurance of older drivers, often stemming from their extensive driving experience and desire for independence, may cause them to hesitate in recognizing limitations imposed by aging-related health factors that affect their driving abilities. This can result in overconfidence and a delay in taking proactive measures to address their challenges. By bridging the gap between subjective confidence and objective impediments, we can reduce the risk of a false sense of security and promote timely decisions for preventive actions while respecting their need for independence and mobility. While numerous prior works have compared older drivers’ self-assessed driving skills to those of drivers in other age groups [22], no study to our knowledge has examined older adults’ self-assessed driving skills specifically in light of the challenges they experience while driving, which we believe as a key contribution of our work.

In the following sections, we propose several potential solutions to close this perceptual gap based on our findings on the challenges and practices older drivers might face. These include fostering a realistic understanding of their capabilities, encouraging open dialogue about aging and safe driving, promoting regular assessments, and increasing the awareness of available resources.

### Foster a Realistic Understanding of Older Drivers’ Capabilities

With age, physiological changes inevitably affect various aspects of driving; however, older drivers may not always recognize these changes or may underestimate their impact on their driving abilities. Therefore, the first step in bridging the gap is to cultivate a realistic understanding among older adults regarding their capabilities and their direct impact on road safety and overall well-being. Understanding their capabilities realistically can allow older drivers to make informed decisions about their driving habits. Furthermore, it empowers them to recognize when adjustments may be necessary, such as limiting driving during certain times of the day or in adverse weather conditions or seeking alternative transportation options when needed. By embracing their limitations and taking proactive steps to address them, older drivers can continue to enjoy the freedom and mobility that driving provides while minimizing the risks associated with age-related changes.

As our findings demonstrated, many older adults may not fully comprehend how age-related changes can directly affect their driving abilities. One way to solve this problem is to provide education tailored specifically for older drivers so that we can emphasize the importance of acknowledging and adapting to age-related changes in a positive and constructive manner. This education can include information about common age-related declines in physical and cognitive functions, such as diminished vision or slower reaction times, and how these changes can affect driving safety.

### Encourage Open Dialogue for Aging and Safe Driving

Families and friends serve as invaluable resources for discussing sensitive topics in positive and constructive ways. In this regard, having an open dialogue for safe driving with families and friends can provide a platform for older adults to openly address their concerns, challenges, and experiences related to aging and driving. Family members and friends can play a crucial role in recognizing the changes in driving abilities and discussing potential solutions, such as driving assessment programs or alternative transportation options.

Open dialogue can take place within various settings, such as community groups, senior centers, or health care facilities, and can involve discussions with peers, family members, and health care professionals. By encouraging open communication, older drivers can gain valuable insights, support, and advice from others who may have faced similar situations or challenges. Moreover, open dialogue can help to destigmatize discussions around aging and driving, breaking down barriers that may prevent older drivers from seeking assistance or support. Many older adults may feel reluctant to acknowledge age-related changes in their driving abilities due to fears of losing their independence or being perceived as incapable [22,42]. By fostering an open and supportive environment, older drivers can feel more comfortable expressing their concerns and seeking guidance without judgment.

In addition, open dialogue promotes education and awareness about the importance of safe driving practices as individuals age. This includes discussions about the potential effects of aging on driving abilities, such as changes in vision, hearing, reaction time, and cognitive function. By raising awareness of these issues, older drivers can better understand the importance of regular self-assessment, professional evaluation, and ongoing monitoring of their driving skills.

### Encourage Regular Assessments

A structured and standardized approach to providing older drivers with realistic insights into their driving abilities and identifying areas that may require improvement or modification can be achieved through regular assessments. This can include assessments of vision, reaction time, cognitive function, and physical mobility. As individuals age, changes in physical and cognitive abilities can occur, which may impact their driving skills. By undergoing periodic evaluations of their driving abilities, older drivers can stay informed about any changes in their capabilities, address emerging challenges proactively, and continue to enjoy the freedom and mobility that driving provides while ensuring the safety of themselves and others on the road.

Furthermore, regular assessments can empower older drivers to stay informed about any changes in their capabilities, take proactive steps to address any emerging challenges, identify potential areas where they may need to adjust their driving habits, and ensure their safety on the road. For example, if an assessment reveals changes in vision or reaction time, older drivers can take measures such as adjusting their driving habits, seeking additional training or support, or even considering alternative transportation options when necessary. Moreover, regular assessments can provide peace of mind for older drivers and their families, knowing that they are actively monitoring their driving abilities and taking steps to maintain their safety [10]. It can also serve as a preventative measure, helping to identify any potential issues before they escalate into more significant concerns.

### Increase the Awareness of Available Resources

There exist useful resources and support tailored to addressing the needs of older drivers with their driving skills and overall health conditions [12,14,15]. For instance, the New York State Office for the Aging has the Older Driver and Pedestrian Safety Project, which offers a handbook for families dealing with the issue of an older driver at risk. The state of New Jersey also provides several resources for older adults who drive, including programs to improve driving skills, assessments to evaluate driving capabilities, and services for drivers with medical conditions. In addition, most other states offer similar programs to support capacity-building initiatives for older drivers. Our findings, however, show limited awareness about available resources and support systems designed to enhance their driving safety and skills. Despite the resources and support systems available for older adults, none of our participants were aware of or had used any of these resources. The lack of awareness about resources can impede their ability to adapt to changing conditions and acquire the knowledge and tools necessary to drive safely as they age.

Increasing the awareness of available resources is crucial for older drivers as it provides them with access to support, information, and services that can enhance their driving safety and overall well-being. Awareness of available resources ensures that older drivers are informed about the various programs and organizations dedicated to promoting safe driving practices among older adults. This includes community-based programs; government agencies; nonprofit organizations; and health care facilities that offer services tailored to the needs of older drivers, such as driver education courses, vehicle safety checks, and mobility assistance programs. By increasing the awareness of these resources, older drivers can proactively seek assistance and support when needed, whether it is addressing age-related changes in their driving abilities, exploring alternative transportation options, or accessing adaptive driving equipment and technologies. This empowers older drivers to take control of their driving safety and make informed decisions that prioritize their well-being.

Moreover, the awareness of available resources helps to combat social isolation and promote social connectedness among older drivers. Many of the resources available to older drivers also provide opportunities for social interaction, peer support, and community engagement, which can have positive effects on mental and emotional well-being. By participating in group activities, support groups, or educational workshops, older drivers can connect with others who share similar experiences and concerns, reducing feelings of isolation and loneliness.

### Limitations

Our findings must be evaluated in light of several limitations. First, our sample size was small, and all participants were recruited from the greater New York area. Second, most participants were considerably healthier than average older adults. We believe this is because older adults with health issues that prevent them from daily driving might have been naturally excluded due to an inclusion criterion requiring them to be actively engaged in driving as part of their daily routine at the time of recruitment. However, since we focused on studying older adults who drive daily, having a healthier participant pool was inevitable. Consequently, our participant pool may not accurately represent the broader aging population. While we believe that our findings offer valuable insights into understanding current practices and perspectives on older adults’ driving practices, further research is necessary to explore how older adults from different sociocultural-technical backgrounds or residing in other regions (eg, rural areas) might perceive this topic differently.

### Conclusions

To many older adults, driving is essential for maintaining mobility and independence for aging in place. The absence of driving can significantly affect the overall quality of life of older adults, which results in declines in both their physical and mental health. Therefore, it is important to ensure that these older drivers prolong their driving skills for independence, healthy aging, and overall quality of later life. This study investigated the daily driving practices of older adults and uncovered a noticeable gap between their perceived confidence in driving abilities and the challenges they encountered on the road. This disparity calls attention to a significant need for support that surpasses the actual assistance currently available to maintain driving performance among older adults. Drawing from our findings, we discussed actionable implications and empirical interventions aimed at sustaining older adults’ driving capabilities. We are hopeful that these suggestions are valuable for practitioners and researchers focused on enhancing overall well-being and quality of life in later stages toward facilitating aging in place.
